# Functional and Structural Characterization of FAU Gene/Protein from Marine Sponge *Suberites domuncula*

**DOI:** 10.3390/md13074179

**Published:** 2015-07-07

**Authors:** Dragutin Perina, Marina Korolija, Marijana Popović Hadžija, Ivana Grbeša, Robert Belužić, Mirna Imešek, Christine Morrow, Melanija Posavec Marjanović, Tatjana Bakran-Petricioli, Andreja Mikoč, Helena Ćetković

**Affiliations:** 1 Division of Molecular Biology, Ruđer Bošković Institute, Zagreb 10000, Croatia; E-Mails: dperina@irb.hr (D.P.); Mirna.Imesek@irb.hr (M.I.); Melanija.Posavec@irb.hr (M.P.M.); 2 Forensic Science Centre “Ivan Vučetić”, Zagreb 10000, Croatia; E-Mail: korolijamm@gmail.com; 3 Division of Molecular Medicine, Ruđer Bošković Institute, Zagreb 10000, Croatia; E-Mails: Marijana.Popovic-Hadzija@irb.hr (M.P.H.); Robert.Beluzic@irb.hr (R.B.); 4 The Mina and Everard Goodman Faculty of Life Sciences, Bar-Ilan University, Ramaty-Gan 5290002, Israel; E-Mail: ivana.grbesa3@gmail.com; 5 Queen’s University Belfast, Marine Laboratory, Portaferry BT22 1PF, Northern Ireland, UK; E-Mail: christinemorrow@gmail.com; 6 Department of Biology, Faculty of Science, University of Zagreb, Zagreb 10000, Croatia; E-Mail: tbakran@biol.pmf.hr

**Keywords:** ribosomal protein genes, snoRNA, FAU, RPS30, SNORA62, evolution, Porifera

## Abstract

Finkel-Biskis-Reilly murine sarcoma virus (FBR-MuSV) ubiquitously expressed (*FAU*) gene is down-regulated in human prostate, breast and ovarian cancers. Moreover, its dysregulation is associated with poor prognosis in breast cancer. Sponges (Porifera) are animals without tissues which branched off first from the common ancestor of all metazoans. A large majority of genes implicated in human cancers have their homologues in the sponge genome. Our study suggests that *FAU* gene from the sponge *Suberites domuncula* reflects characteristics of the *FAU* gene from the metazoan ancestor, which have changed only slightly during the course of animal evolution. We found pro-apoptotic activity of sponge FAU protein. The same as its human homologue, sponge FAU increases apoptosis in human HEK293T cells. This indicates that the biological functions of FAU, usually associated with “higher” metazoans, particularly in cancer etiology, possess a biochemical background established early in metazoan evolution. The ancestor of all animals possibly possessed FAU protein with the structure and function similar to evolutionarily more recent versions of the protein, even before the appearance of true tissues and the origin of tumors and metastasis. It provides an opportunity to use pre-bilaterian animals as a simpler model for studying complex interactions in human cancerogenesis.

## 1. Introduction

The evolution of cancer is still not fully understood. However, it is known that the genes of cellular cooperation that evolved along with multicellularity, when malfunctioned, contribute to cancer development [1]. The majority of these genes are involved not only in cell division and cell growth, but also in cell adhesion, apoptosis, developmental signaling pathways, recognition of self and non-self and differentiation of various cell types. All living animals descended from the 600 million year old common ancestor, including sponges and humans [2]. Sponges are the simplest animals, but despite the absence of tissues, their genomes contain a large majority of genes that have been implicated in human cancer [2]. However, the functions of these genes and biochemical characteristics of their products within the sponge cells remained largely unknown [3,4]. Tumors have not yet been identified in sponges, although computational studies have predicted that most metazoans might be prone to develop tumors [5]. Moreover, naturally occurring tumors have recently been described in two cnidarian species [6]. These findings suggest that understanding of cancer associated genes in simpler animals without tissues and blood vessels could help in understanding the more complex interactions of their homologues in higher Metazoa.

Ribosome assembly is a complex process requiring coordinated activation of more than 200 non-ribosomal factors, including numerous small nucleolar RNAs (snoRNAs). Their role in modification of ribosomal RNAs (rRNAs) was shown to be essential for the correct assembly of the ribosome [7,8]. Three major classes of snoRNAs include: C/D box snoRNAs (primarily guide the 2′-*O*-methylation of target rRNAs), H/ACA box snoRNAs (typically guide pseudouridylation of target rRNAs) and small Cajal-body-specific RNAs (scaRNAs) (typically target snoRNAs). snoRNAs are believed to be the most ancient non-coding RNAs (ncRNAs) [9]. Many examples of ncRNAs displaying both snoRNA and microRNA (miRNA) characteristics suggest a possible evolution from one type to the other [10]. snoRNAs are nonautonomous transposable elements that can gain additional genomic copies, usually by a copy-and-paste mechanism involving an RNA intermediate. These snoRNA retroposons (snoRTs), like Alu element, use the LINE L1 machinery for their mobilization, and probably play an important role in evolution of mammalian genome [11].

Ribosomal proteins (RPs) are an evolutionarily conserved component of ribosome in every cell of every organism. Analysis of 66 complete genomes revealed that 34 RPs are common to all living organisms [12]. Many RPs possess additional extraribosomal functions in cells. They are involved in many processes within the ribosome system, surveillance of ribosome synthesis, but also in replication and regulation of cell growth, apoptosis and cancer [13]. Human *FAU* gene (Finkel-Biskis-Reilly murine sarcoma virus (FBR-MuSV) ubiquitously expressed) encodes the ribosomal protein S30 (RPS30) fused with an ubiquitin-like protein FUBI [14]. Retrovirus FBR-MuSV, which contains genes *v-fos* and *fox*, can induce osteosarcomas in susceptible mice [15]. *Fox* is an antisense sequence to the cellular gene *FAU*, which indicates a putative tumor suppressor role for FAU. Moreover, the *FAU* gene is down-regulated in human breast, prostate and ovarian tumors and its down-regulation is strongly associated with poor prognosis in breast cancer [16,17,18]. Pro-apoptotic regulatory role for FAU has recently been described [19]. Since the failure of apoptosis is fundamental to the development of many cancers, regulation of apoptosis may serve as the functional background for the extraribosomal function of this RP.

Our study suggests that *FAU* gene from the sponge *Suberites domuncula* probably reflects the characteristics of the *FAU* gene from metazoan ancestor that changed only slightly during animal evolution. FAU protein from sponge possesses pro-apoptotic activity and increases apoptosis in human HEK293T cells, same as its human homologue. This suggests that biological functions of FAU, usually associated with the origin of cancer in “higher” metazoans, possess biochemical background established early in metazoan evolution. Our results implicate that the ancestor of all animals possessed FAU protein with the structure and the function similar to its evolutionarily recent versions, even before the appearance of true tissues and the origin of tumors and metastasis.

## 2. Results and Discussion

### 2.1. Structure and Evolution of Metazoan FAU Gene

**Figure 1:**
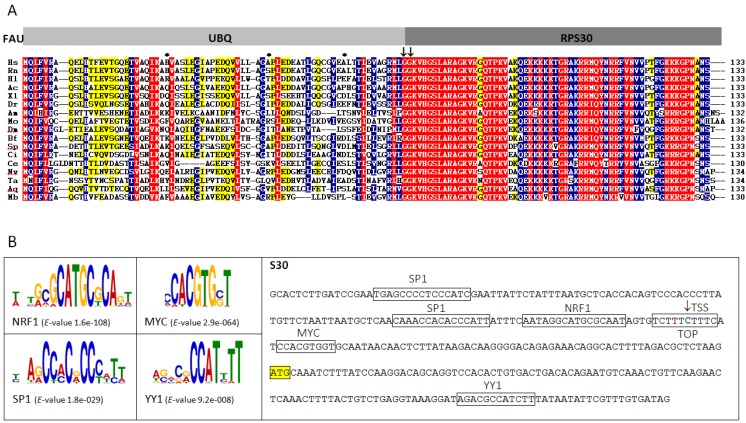
(**A**) Multiple sequence alignment of FAU proteins from representative species (*Homo sapiens* (Hs), *Rattus norvegicus* (Rn), *Haliaeetus leucocephalus* (Hl), *Anolis carolinensis* (Ac), *Xenopus laevis* (Xl), *Danio rerio* (Dr), *Apis mellifera* (Am), *Metaseiulus occidentalis* (Mo), *Drosophila melanogaster* (Dm), *Branchiostoma floridae* (Bf), *Strongylocentrotus purpuratus* (Sp), *Ciona intestinalis* (Ci), *Caenorhabditis elegans* (Ce), *Nematostella vectensis* (Nv), *Trichoplax adhaerens* (Ta), *Amphimedon queenslandica* (Aq), *Monosiga brevicollis* (Mb)). G–G dipeptide motif and missing lysine residues which serve as sites for polyubiquitin chain formation are marked with arrows and asterisks, respectively; red = 100%, blue = 80% and yellow = 60% identity; (**B**) Over-represented motifs in sponge ribosomal protein gene (RPG) promoters and promoter sequence of sponge *FAU* with indicated translational start site (marked with yellow rectangle), transcription start site (TSS, marked with arrow) and transcription factor binding sites and terminal 5′-terminal oligopyrimidine (TOP) tract (marked with rectangles).

*FAU* gene encodes an ubiquitin-like protein (FUBI) fused to the ribosomal protein S30 (RPS30). This type of gene organization seems to be conserved throughout metazoan evolution (Figure 1A). Sponge FAU protein has 61% amino acid sequence identity (78% sequence similarity) with human FAU and is more similar to its homologues from human or rat, than to those from invertebrates *Caenorhabditis elegans* (47% sequence identity, 62% similarity) and *Drosophila melanogaster* (50% sequence identity, 68% similarity). Both invertebrate model organisms underwent recent accelerated evolution [20]. Since sponge FAU changed only slightly during metazoan evolution, it seems to be a promising candidate to effectively reflect FAU protein structure of the ancestral metazoan ribosome. It is also noteworthy that FUBI domain is much less conserved than RPS30. However, G–G dipeptide motif, which participates in bond formation between ubiquitin and lysine residues of target proteins, is conserved in all analyzed metazoans (Figure 1A). As already observed in human FAU, none of the lysine residues which serve as sites for polyubiquitin chain formation, are present in any of the analyzed FAU homologues (Figure 1A). This indicates that FUBI is unlikely to have an analogous role to ubiquitin in protein degradation and that this diverse role was probably established early in metazoan evolution.

Synchronized expression of ribosomal protein genes (RPGs), driven by the promoters of similar strength, ascertains equimolecular amount of ribosomal proteins in the cell, which is prerequisite for the ribosome assembly [21,22]. Human over-represented motifs in RPG promoters are identified as targets for YY1, NRF1, c-Myc and SP1 transcription factors [21]. The 5′-terminal oligopyrimidine (TOP) tract in human RP transcripts is also known to be essential for the control of gene expression, at both the transcriptional and the translational levels [23,24]. To test which elements are present in the *FAU* promoter, we compared over-represented motifs in RPG promoters of the sponge *Amphimedon queenslandica* with *FAU* sequence. In the majority of analyzed human RPG promoters, YY1 motifs were found downstream of the transcription start site (TSS) [25]. This motif is also present downstream in the sponge *FAU* (*p =* 3.00 × 10^−7^) (Figure 1B). Two SP1 and one NRF1 binding sites were found upstream of the TSS (*p =* 4.08 × 10^−7^, *p =* 1.39 × 10^−7^, *p =* 7.10 × 10^−7^, respectively), while E-box palindromic core CACGTG was also identified in *FAU* promoter (*p =* 3.12 × 10^−6^). Among many transcription factors that recognize the E-box, oncoprotein c-Myc (MYC) is known to bind to it [26]. c-Myc enhances ribosomal biogenesis by up-regulating transcription mediated by all three RNA polymerases [27]. This implies possible participation of the FAU protein in the analogous c-Myc-enhanced ribosomal biogenesis established early in metazoan evolution. The TSS was identified in TOP (Figure 1B). The TOP motif has a dual regulatory function in vertebrates. It is a part of a *cis*-regulatory element in transcriptional regulation and has a role at the translational level [23,24]. In certain physiological conditions, it inhibits binding of the translational regulatory proteins or the translational machinery to the mRNA [28]. The presence of TOP and all other elements in sponge *FAU* promoter indicates that the foundations of the human *FAU cis*-regulation were established early in metazoan evolution.

**Figure 2:**
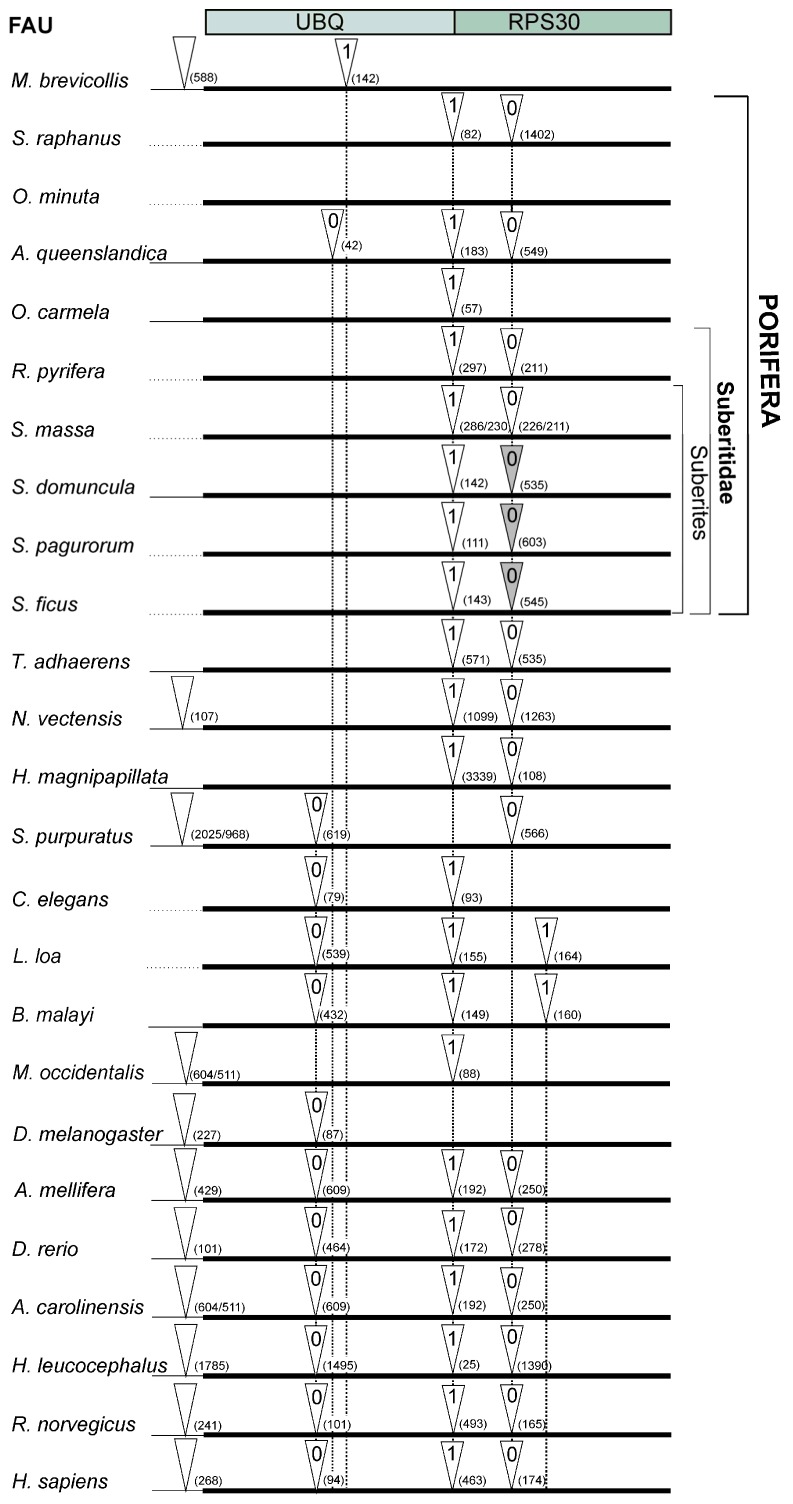
Intron-mapping of *FAU* genes from representative species. White triangles indicate positions of the introns and gray triangles indicate presence of SNORA62. The number within triangle denotes the intron phase and the number in brackets intron length. The thin line indicates the 5′ UTR region.

Multiple sequence alignment of *FAU* gene orthologues was used to characterize intron dynamics by comparing the intron positions (Figure 2). Most of the positions, phases, and the number of introns in *FAU* genes were not significantly changed from sponge to human (Figure 2). The most conserved intron position separates the UBQ from RPS30 domain in *FAU* and varies from 25 to 3339 bp. The average value of sponge RPG intron length is 164 bp [29], while human RPGs have significantly longer introns, with the average length of 760 bp [30]. Transposable element insertions play an important role in the evolution of intron size [31]. Therefore, we searched for over-represented elements in *FAU* introns.

### 2.2. Identification of snoRNAs in FAU Introns of Sponges

**Figure 3:**
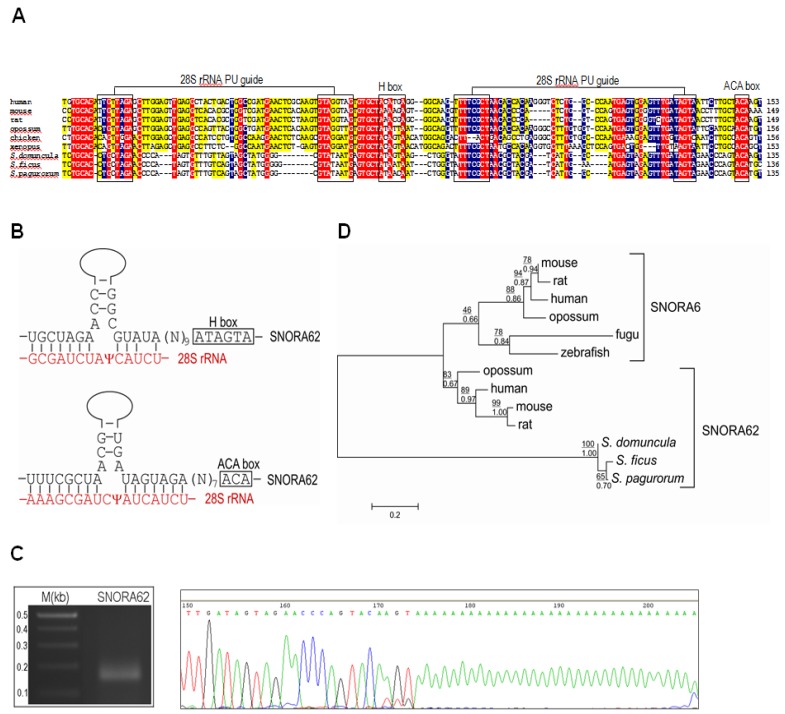
(**A**) SNORA62 conserved in the last introns of the *FAU* gene in *S. domuncula*, *S. ficus* and *S. pagurorum*. All essential snoRNA elements are conserved and a putative pseudouridylation (PU) guide site is designated; red = 100%, blue = 80% and yellow = 60% identity; (**B**) Target sites of SNORA62 are conserved in sponges and marked with psi; (**C**) Experimental verification of transcription of sponge SNORA62. Polyadenylated snoRNAs were amplified, cloned and sequenced; (**D**) Maximum likelihood (ML) phylogenetic tree of snoRNAs from representative species. Bootstrap values for ML are given above and MCMC below the line. The scale bar indicates the genetic distance of the branch lengths.

The accumulating genomic data strongly confirm the tendency of snoRNAs to colonize RPGs and ribosome related genes in eukaryotes [32]. The total set of 16 introns in *FAU* gene from eight sponges produced four candidate H/ACA snoRNAs. After a more detailed analysis, we were able to identify three snoRNAs that match a sequence motif of known snoRNAs available on Rfam, the snOPY database and the snoRNA-LBME database. This snoRNA is the sponge orthologue of the human SNORA62 (E2) (Figure 3A). SNORA62 is predicted to guide the pseudouridylation of uridine residues at the position 3830 and 3832 in human 28S rRNA [33]. This target sequence of 28S rRNA is highly conserved between human and analyzed sponges (Figure 3B). The sponge SNORA62 pseudouridylation guide sequence, as well as the H- and ACA-boxes, are also well conserved (Figure 3A). Its expression was verified experimentally (Figure 3C). Phylogenetic analysis of SNORA62/SNORA6 is presented in Figure 3D. The SNORA62 and SNORA6 share the same host gene in vertebrates and SNORA6 probably arose by *cis*-duplication of SNORA62. The SNORA62 homologues from sponges clearly group together and reflect the characteristics of snoRNA ancestral to this snoRNA family, before the duplications and diversifications within the metazoan lineage. snoRNAs are mobile genetic elements, often transferred through retrotransposition, and can therefore participate in diversification and enrichment of transcriptomes through various mechanisms such as intron/exon gain/loss [34]. It is known that snoRNAs can change their genomic location within relatively short vertebrate evolutionary time scales [35]. Since all of examined RPG intron positions were conserved in three *Suberites* species, it was presumed that on this shorter evolutionary time scale, mobility of snoRNAs is not a significant factor that determines intron dynamics [29]. However, we found that within the same genus (*Suberites*), SNORA62 can change its genomic location. In *S. pagurorum*, *S. domuncula* and *S. ficus* this snoRNA is present in the same intron, but apparently is missing in the same conserved intron in *S. massa* (Figure 2). Our results indicate that RPG introns are more dynamic than previously documented and that mobility of snoRNAs plays an important role in RPG (and *FAU*) evolution even on shorter evolutionary time scales.

The other snoRNA shows stable secondary structure with conserved snoRNA parts (Figure S1). In the first intron of FAU gene of the sponge *Rhizaxinella pyrifera*, a single snoRNA with a potential target rRNA was found (Figure S1). This target is not conserved in humans nor has it yet been described as a pseudouridylation site, so we can only speculate about the possible function of this snoRNA.

### 2.3. Subcellular Localization of Sponge FAU

**Figure 4:**
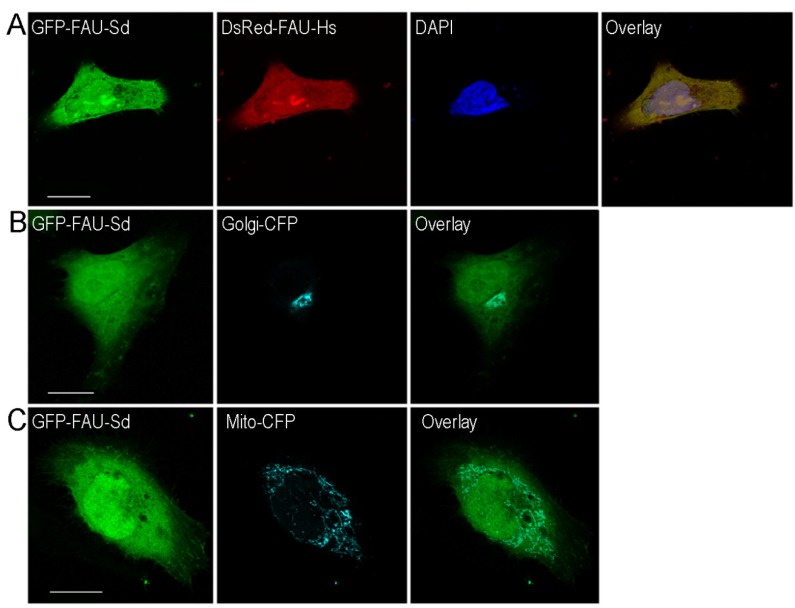
(**A**) Subcellular localization of sponge and human FAU. HeLa cells transiently transfected with sponge (Sd) pEGFP-FAU (green fluorescence), human (Hs) pDsRed-FAU (red fluorescence); (**B**) pECFP-Golgi (cyan) and (**C**) pECFP-mitochondria (cyan). The overlay (yellow) shows colocalization of the human and sponge homologs in panel **A**. Scale bar = 10 μm.

RPs are mainly cytoplasmic, being incorporated into translating ribosomes, but they can be found, at least transiently, in the nucleus [36]. As mentioned before, RPs have non-ribosomal functions when they are off-ribosomal subunits. We transiently transfected HEK293T and HeLa cells with GFP-FAU from *S. domuncula* and DsRed-FAU from human and analyzed the cells 48 hours post transfection using confocal laser scanning microscopy. Proteins show the same localization pattern in both cell types. Both human and sponge proteins exhibit the same subcellular localization (Figure 4A). Proteins are dispersed throughout the cytoplasm rather than being associated with a specific cytoplasmic organelle (Figure 4B,C). Although the signals are present mainly in the cytoplasm, portions can also be clearly observed in the nuclei (Figure 4A). This result matches the finding of the previous study, where subcellular localization of *Drosophila* RPS30 was analyzed [36]. It has been observed that a fraction of RPS30 is localized in the nucleus and is associated with transcription sites. We presume that, according to cellular localization, extraribosomal functions of FAU at transcription sites were already present in ancestor of all metazoans and remained conserved throughout the evolution.

**Figure 5:**
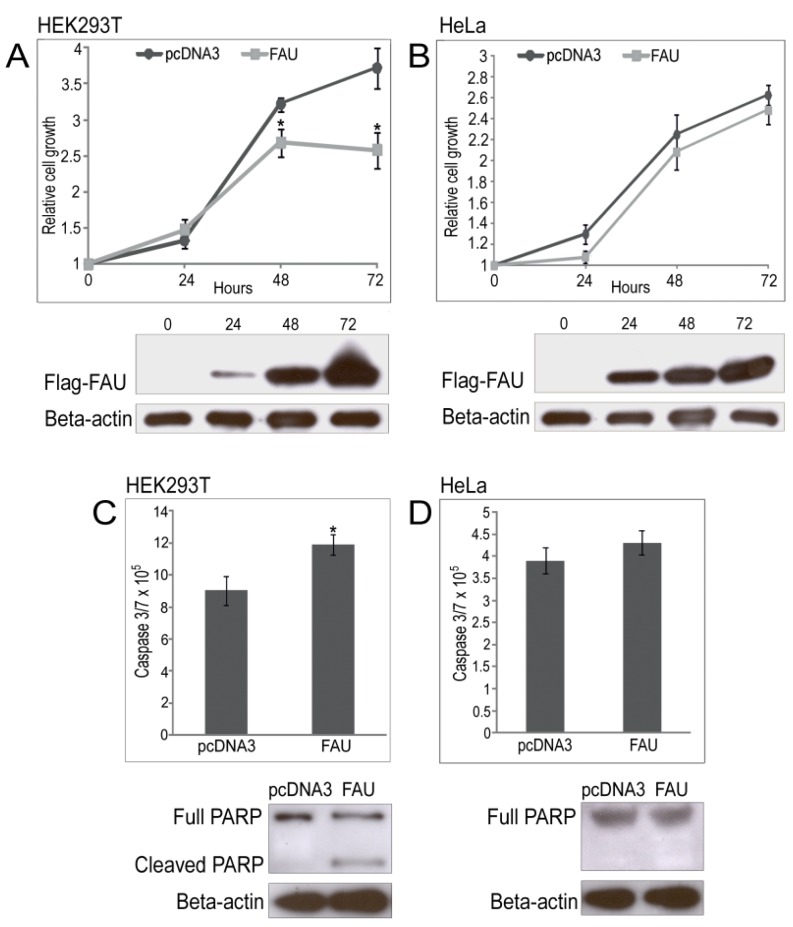
Protein blot validation of sponge Flag-FAU expression and the relative growth rates assessed by CellTiter-Glo assay of HEK293T (**A**) and HeLa cells (**B**); Activities of caspase were measured by a Caspase-Glo 3/7 assay kit and by cleavage of caspase substrate PARP in HEK293T (**C**) and HeLa cells (**D**) transfected with pcDNA3-FAU or empty vector pcDNA3. Data are representative of three independent experiments (* *p <* 0.05).

### 2.4. Overexpression of Sponge FAU in Human Cell Lines

Transient transfection with human pcDNA3-FAU increased apoptosis and decreased short-term cell viability in HEK293T, Jurkat and CEM cell lines [19]. To test whether these functions of FAU protein were established early in metazoan evolution, we transfected HEK293T and HeLa cell lines with sponge pcDNA3-FAU. The growth rate of cells was evaluated at different time points after transfection, to determine whether overexpression of sponge FAU affects cell proliferation. Transfection markedly increased Flag-FAU levels (Figure 5A,B). Empty vector pcDNA3 was used as control. Subsequently, the proliferation of pcDNA3-FAU-transfected HEK293T cells was decreased 1.44-fold (*p* = 0.008) when compared to cells with pcDNA3 control (Figure 5A). Cell viability decreased by 30.1% 72 h post-transfection compared to cells possessing the control vector. Interestingly, these effects were not observed in HeLa cancer cell line (Figure 5A). To further investigate the mechanism underlying the observed, we measured caspase-3 and -7 activities in transfected cell lines. pcDNA3-FAU-transfected HEK293T cells show 1.31-fold (*p* = 0.014) increased caspase activity as compared to pcDNA3 transfected cells (Figure 5C). However, apoptotic cells were not elevated in HeLa cell line, and neither the cleavage of PARP, caspase-3 substrate, was observed (Figure 5D). Interestingly, similar results were obtained when human Flag-FAU was overexpressed in HeLa cells (Figure S2). Our results indicate that pro-apoptotic activity of FAU was established early in metazoan evolution, but appears to be cell type specific as its expression leads to efficient apoptosis in HEK293T but not in HeLa cell line. Since both human and sponge FAU do not activate caspases in HeLa cells significantly, but both activate caspases in HEK293T cells, we believe that the mechanism underlying this effect was already present in metazoan ancestor. It was proposed that Bcl-G plays an essential down-stream role in mediating the pro-apoptotic activity of FAU [19]. The molecular mechanisms of apoptosis regulation by FAU remain to be elucidated, but possibly include FUBI-mediated targeting of Bcl-G and/or modulation of the interaction of Bcl-G with other constituents of the cellular apoptotic machinery (caspases for example) [19]. Although down-regulation of Bcl-G inhibits apoptosis induced by FAU, basal apoptosis rates are higher in certain cellular contexts [19]. However, this effect has not been observed in human breast cancer and prostate cell lines [16,17]. This indicates that in certain cell lines, Bcl-G can have anti-apoptotic effects, in addition to the pro-apoptotic activity. Moreover, mouse Bcl-G is expressed in a range of epithelial, as well as in dendritic cells and its loss does not appear to affect any of these cell types [37]. This intriguing contrast in the effects of FAU/Bcl-G on cell fate in different cellular contexts presents attractive possibilities for the development of novel therapies for cancers and could be used for targeted elimination of cancer cells.

From all presented data we can conclude that the structure and the functions of the recent *FAU* gene were established early in metazoan lineage. Tumor suppressor gene function of FAU probably arose before tumor appearance. As sponges do not possess tissues and organs, formation of tumors within them is unlikely. It seems that tumors evolved in parallel with the development of true tissues and organs, yet the extraribosomal functions of RPs were probably established in the metazoan ancestor well before the Cambrian explosion, *i.e.*, before the appearance of diverse groups of multicellular animals. Other highly sophisticated extraribosomal functions of RPs, involved in the origin of cancer and metastasis, evolved together with the appearance of different cell types, tissues and organs. Whereas synchronized *cis*-regulation of RPGs needs to be conserved for equimolecular presence of RPs in the metazoan cell, the evolution of RPGs together with the extraribosomal functions of RPs on shorter evolutionary time scale is probably driven by snoRNAs. snoRNAs can promote, but also suppress tumor development [38]. Numerous studies already provided evidence for the functional importance of snoRNAs in cancerogenesis [39,40]. Our results show that SNORA62 is found in *FAU* gene in the subset of sponges from the genus *Suberites*, which suggests its dynamic and fast evolving nature. This snoRNA is located in the human ribosomal protein gene *SA* (*RPSA*). Both host RPGs possess an extraribosomal function involved in the maintenance of cellular viability through the caspase-dependent regulation of apoptosis [19,41]. Interestingly, *SNORA62* is the most upregulated gene in diallyl sulfide (DAS)-induced apoptosis in HeLa cells, as a part of p53, mitochondria- and caspase- pathways [42]. It would be interesting to test whether this snoRNA can act as an oncogene like SNORA42 [43], or tumor suppressor like U50 snoRNA [44] and if these functions interfere and modulate similar functions of its host genes. The number of ncRNA is exceedingly lower in basal metazoans than in “higher” animals, which may indicate that metazoan complexity correlates with an increasing number of ncRNAs [45]. If SNORA62 can modulate apoptotic effect of its host genes, it will provide new strategies for targeting FAU. In the longer term, the development of techniques for the manipulation of snoRNAs may also be productively directed towards the manipulation of genes involved in cancerogenesis for cancer therapy. Analyses of sponge FAU gene presented in our paper provides the opportunity to determine direction for further investigation of FAU and evaluate its potential for cancer treatment. Undoubtedly, sponges could be used as an informative model in cancer research, not only in terms of targeting genes involved in cancerogenesis by snoRNAs, but also in targeting snoRNAs themselves. In order to evaluate their potential for cancer treatment, further investigation of highly conserved and mobile snoRNAs found in the metazoan common ancestor is needed.

## 3. Experimental Section

### 3.1. Materials

Live specimens of the sponge *Suberites domuncula* (Porifera, Demospongiae, Tetractinomorpha, Hadromerida, Suberitidae) and *Sycon raphanus* (Porifera, Calcarea, Calcaronea, Leucosolenida, Sycettidae) were collected in the Northern Adriatic Sea near Rovinj, Croatia, and stored at −80 °C. Specimens of *Oopsacas minuta* (Porifera, Hexactinellida, Hexasterophora, Lyssacinosida, Leucopsacidae) were collected in the cold water pit in the Middle Adriatic and specimens of *Rhizaxinella pyrifera* (Porifera, Demospongiae, Tetractinomorpha, Hadromerida, Suberitidae) were collected in the Middle Adriatic Pit, around 200 m depth and stored in 96% EtOH. *Suberites ficus* and *Suberites pagurorum* were collected in Ireland, while *Suberites massa* in the English Channel.

### 3.2. Sequence Analyses

Homology searches and sequence retrievals were done using BLAST (NCBI, NIH, Bethesda, MD, USA: http://www.ncbi.nlm.nih.gov). Sequences were analyzed using Lasergene (DNAStar, Madison, WI, USA). Multiple sequence alignments (MSA) were performed with CLUSTALX [46]. The exact position and the phase of each intron were verified by manual inspection and statistical data were extracted from GeneDoc (http://www.psc.edu/biomed/genedoc).

Over-represented motifs were searched using MEME [47], as described earlier [22]. Statistical significance of the motif (*E*-value) and *p*-value (statistical significance of predicted sites in *FAU*) were extracted from MEME. The location of transcription factor binding sites relative to the transcription start site was also extracted from MEME results for *FAU* sequence within a set of sequences with the same identified motif. Obtained motifs were then compared using TOMTOM [48] against the TRANSFAC database of known motifs [49].

### 3.3. Isolation of Genomic DNA and Sequencing of FAU Gene

For genomic DNA preparation, sponge specimens were cut into pieces, frozen in liquid nitrogen and grounded to fine powder from which total DNA was isolated using the Genomic DNA Purification kit (QIAGEN, Redwood City, CA, USA). Sponge *FAU* gene was amplified by PCR, using LA Taq DNA polymerase (Takara, Japan) and primers specific for ends of the coding sequence of: Demospongiae (5′-GCAAGTTTTCGTACAAGGAGGGGC-3′ and 5′-GGTGCATTTGAGTTTGGTCCAC-3′), Hexactinellida (5′-ATGCAAGTGTTTGCACAAAC-3′ and 5′-CTAACTAGCTGAGTTTGAG-3′) and Calcarea (5′-CAGATTTTCATTCAGGCCCAGGC-3′ and 5′-GCGTTAGAGTTGGGTC-3′). The amplified fragments were separated on a 0.8% agarose gel, purified and cloned into the pGEM-T vector (Promega, Madison, WI, USA). Positive clones were sequenced using T7/pUC primers.

### 3.4. Isolation and Characterization of Small RNAs

snoSeeker was used to identify snoRNAs in introns of *FAU* genes [50]. Rfam [51], snOPY (http://snoopy.med.miyazaki-u.ac.jp/) and the snoRNA-LBME databases [52] were used to check whether snoRNAs match to any of the known snoRNAs’ motifs. The secondary structure of snoRNA was computed using the RNAfold program of the Vienna RNA Package [53]. Isolation of small RNAs from sponge was described previously [29]. In brief, fresh specimens of *S. domuncula* were cut into pieces, frozen in liquid nitrogen and grounded to a fine powder. mirPremier microRNA Isolation Kit (Sigma, St. Louis, MO, USA) was used, according to the manufacturer’s protocol for plant tissue. Polyadenylation was performed by incubation with *E. coli* Poly(A) Polymerase (BioLabs). Poly(A) tailing reaction mixture was then reverse transcribed using the SuperScript II Reverse Transcriptase (Invitrogen, Waltham, MA, USA) and a modified poly-d(T) primer (5′-GCGTAAGTGACTAGCGTGTTTTTTTTTTTTVN-3′). The resulting cDNA was used for PCR with a reverse primer (5′-GCGTAAGTGACTAGCGTG-3′) and forward primer specific for predicted SNORA62 (5′-CCCCATAGTGTTTGTTAGTAGC-3′). The product was cloned into the pGEM-T vector (Promega). Positive clones were sequenced using the ABI PRISM BigDye Terminator v3.1 Ready Reaction Cycle Sequencing Kit and T7/pUC primers.

For phylogenetic analyses, multiple alignments were performed with CLUSTALW Ver. 1.6 [54]. Aligned sequences were imported into MEGA version 6 [55], and analyzed by Maximum Likelihood (ML), while the Bayesian MCMC analysis was conducted in MrBayes v. 3.1.2. [56]. Bootstrap tests were performed with 1000 replicates. The model for ML analysis was selected using Modeltest 3.7 and the Akaike Information Criterion (AIC) [57], which indicated Hasegawa-Kishino-Yano model (I + G) [58].

### 3.5. Cell Culture

Human HeLa cell line (ATCC) and HEK293T cell line were cultured in l-glutamine DMEM (Invitrogene) supplemented with 10% fetal bovine serum (FBS, Invitrogene) and GA-1000 (30 mg/mL Gentamicin and 15 µg/mL Amphotericin) (Lonza, Basel, Switzerland) at a 1:1000 ratio in humidified chamber with 5% CO_2_ at 37 °C.

### 3.6. Plasmid Constructions

Eukaryotic expression vector pcDNA3 (Invitrogen) was digested with BamHI and EcoRI. The insert containing the FLAG sequence and sponge FAU was cloned using these sets of primers: 5′-GTCTAGGGATCCACGAGATGGACTACAAGGACGACGACGATAAGATGCAAGTTTTCGTA-3′ and 5′-CTAGACGAATTCTCATCACTGAGGTGCATTTG-3′, and human FAU using: 5′-GTCTAGGGATCCACGAGATGGACTACAAGGACGACGACGATAAGATGCAGCTCTTTGTC-3′ and 5′-CTAGACGAATTCTTATTAAGAGTTGGCATTGG-3′ For localization assay, sponge FAU was cloned in fusion with GFP using pEGFP-C1 (XhoI/BamHI restriction sites) with the following set of primers: 5′-CCACTCGAGCTATGCAAGTTTTCGTACAAGG-3′ and 5′-GACGGATCCTCATCACTGAGGTGCATTTG-3′, and human using pDsRed-C1 with 5′-CCACTCGAGCTATGCAGCTCTTTGTCCGCGC-3′ and 5′-ACAGGATCCTTAAGAGTTGGCATTGGGGCCC-3′ set of primers.

### 3.7. Transient Transfections and Laser Scanning Confocal Microscopy

Lipofectamine 3000 reagent (Invitrogen) was used for HeLa cells transfections, according to the manufacturer’s instructions. Twenty four hours before transfection 5 × 10^4^ cells were seeded onto 24-well culture slides containing DMEM supplemented with 10% FBS to obtain 80% confluence. Cells were transfected with 500 ng of plasmid DNA. Forty eight hours post transfection, the cells were washed with PBS pH 7.5, fixed in 4% formaldehyde, and mounted in SlowFade Diamond Antifade Mountant with or without DAPI (Molecular Probes, Waltham, MA, USA).

Fluorescent images were obtained by Leica SP8 X FLIM laser scanning confocal microscopy equipped with HC PL APO CS2 63×/1.40 OIL objective. GFP was excited by 488 nm laser line, CFP using 433 nm, DAPI using 405 nm and DsRed at 560 nm laser lines.

### 3.8. Immunoblotting

The cell lysates were loaded on SDS-PAGE and electrotransferred to a PVDF Hybond-P membrane (Amersham Biosciences, Piscataway, NJ, USA). The membranes were incubated with anti-FLAG M2 antibody (1:5000) (Sigma) for detection of FLAG-FAU or anti-PARP antibody (1:5000) (ab137653). Protein bands were visualized using chemiluminescence detection (Amersham ECL Plus, GE Healthcare, Parramatta, Australia).

### 3.9. Cell Viability Assay

HeLa cells were transfected as described above, trypsinized 24 h post-transfection and 10^3^ viable cells suspended in 100 µL 10% FBS DMEM were added to each well of 96-well plates. HEK293T cells were transfected using standard calcium phosphate method [59]. Cell proliferation was measured using CellTiter-Glo Luminescent Cell Viability Assay (Promega) according to the manufacturer’s instructions with luminometer Infinite 200 (TECAN). Concentration of ATP was measured in triplicate.

### 3.10. Apoptosis Assay

Cells were transfected as described above, trypsinized 24 h post-transfection and 5 × 10^3^ viable cells suspended in 100 µL 10% FBS DMEM were seeded in 96-well culture plates. Caspase-3/7 activity was measured using the Caspase-Glo 3/7 Assay Kit (Promega) according to the manufacturer’s instructions with luminometer Infinite 200 (TECAN).

### 3.11. Statistical Analyses

Statistical analysis was performed using Student’s *t*-test. Probabilities of less than 0.05 were considered statistically significant.

## Supplementary Files

Supplementary File 1
