# Supplementary material for: Functional and Structural Characterization of FAU Gene/Protein from Marine Sponge Suberites domuncula
Source: Mar Drugs. 2015 Jul 7;13(7):4179–96. doi: 10.3390/md13074179 (PMC4515611; doi:10.3390/md13074179)
Supplement: Supplementary File 1 [file marinedrugs-13-04179-s001.docx]

Supplementary Information


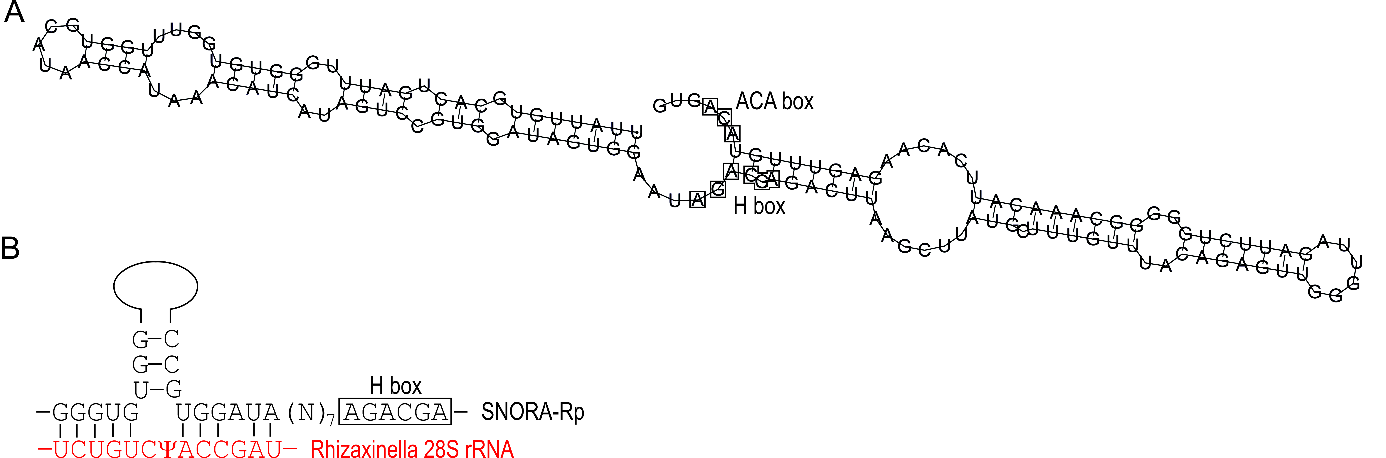


**Figure S1.** (**A**) Secondary structure of a novel putative H/ACA snoRNA found in the first intron of the *FAU* gene in *R. pyrifera* and (**B**) a potential 28S rRNA target site.


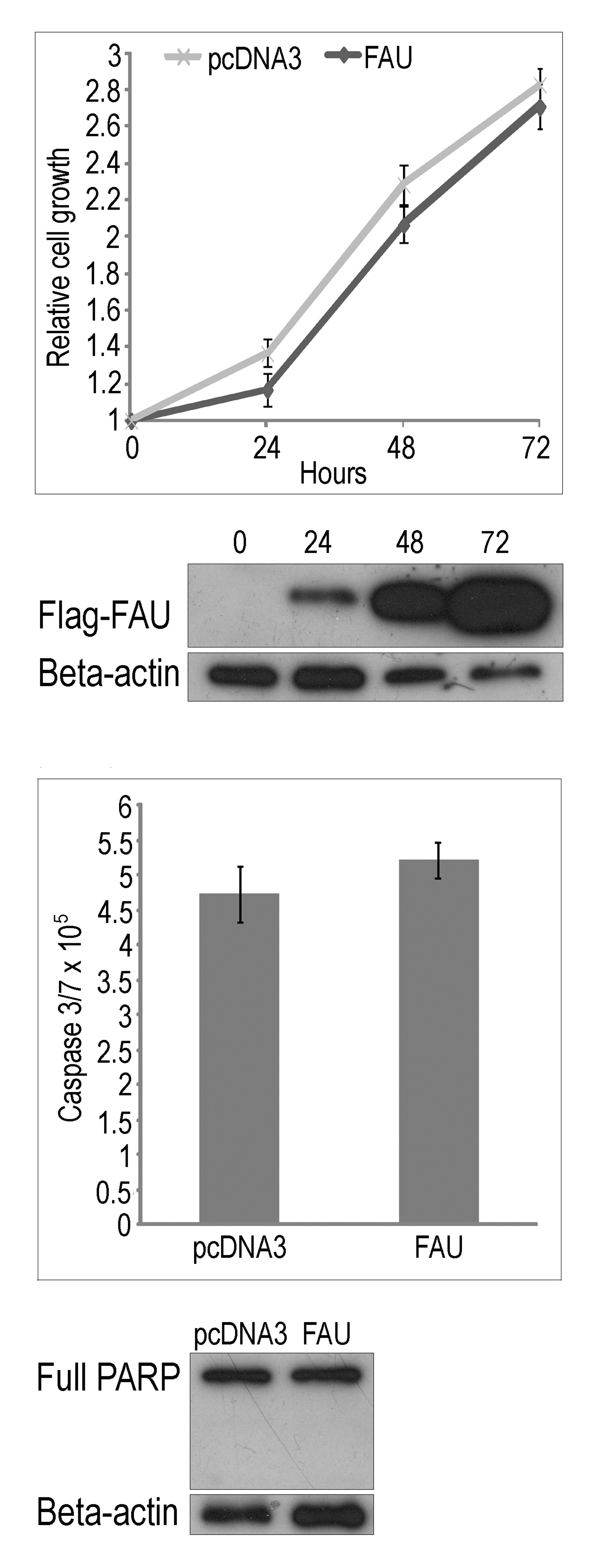


**Figure S2.** Protein blot validation of human Flag-FAU expression and the relative growth rates assessed by CellTiter-Glo assay of HeLa cells. Activities of caspase were measured by a Caspase-Glo 3/7 assay kit and by cleavage of caspase substrate PARP in HeLa cells transfected with pcDNA3-FAU or empty vector pcDNA3. Data are representative of three independent experiments.
